# DrugCombo: an informatics bridge for anticancer drug combination Phase I trial design

**DOI:** 10.1093/database/baaf043

**Published:** 2025-09-24

**Authors:** Lei Wang, Shijun Zhang, Lai Wei, Yuxi Zhu, Naleef Fareed, Lijun Cheng, Williams Carson, Dwight Owen, Yong Zang, Suyu Liu, Aditi Shendre, Xiaofu Liu, Lang Li

**Affiliations:** Department of Biomedical Informatics, The Ohio State University College of Medicine, 1800 Cannon Drive Columbus, OH 43210, United States; Department of Biomedical Informatics, The Ohio State University College of Medicine, 1800 Cannon Drive Columbus, OH 43210, United States; Department of Biomedical Informatics, The Ohio State University College of Medicine, 1800 Cannon Drive Columbus, OH 43210, United States; Center for Biostatistics, The Ohio State University College of Medicine, 1800 Cannon Drive Columbus, OH 43210, United States; Department of Pediatrics, University Hospitals Rainbow Babies & Children’s Hospital, 2101 Adelbert Rd, Cleveland, OH 44106, United States; Department of Biomedical Informatics, The Ohio State University College of Medicine, 1800 Cannon Drive Columbus, OH 43210, United States; Department of Biomedical Informatics, The Ohio State University College of Medicine, 1800 Cannon Drive Columbus, OH 43210, United States; Division of Surgical Oncology, The Arthur G. James Cancer Hospital and Richard J. Solove Research Institute, Comprehensive Cancer Center, The Ohio State University, 410 W. 10th Ave, Columbus, OH 43210, United States; Department of Surgery, Comprehensive Cancer Center, The Ohio State University, 410 W. 10th Ave, Columbus, OH 43210, United States; Division of Medical Oncology, Department of Internal Medicine, The Ohio State University Comprehensive Cancer Center, 1800 Cannon Drive, Columbus, OH 43210, United States; Center for Computational Biology and Bioinformatics, Indiana University School of Medicine, 410 W. 10th Street, Indianapolis, IN 46202, United States; Department Biostatistics and Health Data Sciences, Indiana University School of Medicine, 410 W. 10th Street, Indianapolis, IN 46202, United States; Department of Biostatistics, The University of Texas MD Anderson Cancer Center, 7007 Bertner Avenue, Houston, TX 77030, United States; Department of Biomedical Informatics, The Ohio State University College of Medicine, 1800 Cannon Drive Columbus, OH 43210, United States; Department of Biomedical Informatics, The Ohio State University College of Medicine, 1800 Cannon Drive Columbus, OH 43210, United States; Department of Biomedical Informatics, The Ohio State University College of Medicine, 1800 Cannon Drive Columbus, OH 43210, United States

## Abstract

The promise of combinational drug therapies for cancer is hindered by the high failure rate of Phase I trials, perhaps attributable to the inavailability of an integrated source of toxicity data for cancer drugs to aid clinicians and biostatisticians designing trials. To this end, we developed DrugCombo, a knowledge base that integrates drug toxicity along with other data for single drugs and drug combinations from various sources. We extracted drug toxicity data from drug labels using the Microsoft Research Bidirectional Encoder Representations from Transformers for Biomedical Text Mining (MSR BiomedBERT) and manually from PubMed. DrugCombo is the first such database to contain crucial data, such as maximum tolerable dose (MTD), dose-limiting toxicity (DLT), and dose range, for Phase I clinical trial design as well as pharmacokinetics evidence of drug interaction among cancer drugs. Currently, DrugCombo has integrated 8797 drug interactions from DrugBank; 3995 severe adverse drug events (ADEs) and 95 535 common ADEs from drug labels; 1 816 030 ADEs from United States Food and Drug Administration Adverse Event Reporting System; and MTD and DLTs from 2592 Phase I trials. Using these data, we retrospectively investigated a Phase I trial of axitinib and nivolumab in 12 patients. Exploring the toxicity profile of each drug, we recognized that the initial study design may have overlooked overlap toxicity between them, leading to possibly excessive starting doses and dose ranges of axitinib and nivolumab. DrugCombo provides a comprehensive resource of toxicity data that will assist researchers designing Phase I trials in selecting appropriate starting doses and dose ranges for successful outcomes.

**Database URL**: http://drugcombo.info/

## Introduction

Combination therapies targeting multiple tumour mechanisms are more efficacious than single-agent therapies in most patients with cancer, and ClinicalTrials.gov [1] data reflect an increased number of trials of cancer drug combinations from 905 in 2007 to 1844 in 2023. Unfortunately, a 58% failure rate [2] of Phase I clinical trials poses a challenge to the development of cancer drugs for clinical use.

A Phase I trial properly designed based on a comprehensive assessment of all available data regarding each drug. IDrug’s mechanisms and interactions with paired drugs could accelerate the translational process of drug combinations from the preclinical phase to the Phase II/III setting, allowing safe treatment within the proper dose range and ultimately minimizing patient exposure to severe or life-threatening adverse drug events (ADEs). In the last several decades, the biostatistics community has developed many innovative Phase I trial designs [3, 4], such as the continual reassessment (CRM) and Bayesian optimal interval (BOIN) methods [5], to improve operating characteristics over those of the standard ‘3 + 3’ dose-escalation design. Nevertheless, regardless of their strength, the success of statistical designs relies on accurate prior knowledge of single-drug or drug-combination dose-limiting toxicities (DLTs), maximum tolerated doses (MTDs), and potential pharmacokinetic (PK) drug–drug interactions (DDIs).

Our in-depth review of existing databases of single- and combinational-drug PK and toxicity information (Table 1) revealed that no single database integrates both toxicity and PK data. Rather, drug toxicity data are reported in the United States Food and Drug Administration (FDA) Adverse Event Reporting System (FAERS) [6] and drug labels [7]. PK data are distributed in the Drug Combination Database (DCDB) [8], Certera Drug Interaction Database (DIDB^®^) [9], DrugBank [10], Transportal drug transporter database of the University of California, San Francisco in collaboration with the FDA [11], Transformer database of the National Center for Biotechnology Information (NCBI) [12], and the Health and Human Services (HHS) Pharmacogenomics Knowledgebase (PharmGKB^®^) [13]. Important DLT and MTD data are fragmented and dispersed across numerous publications and data sources, lacking systematic curation and organization.

**Table 1.** Existing drug databases and data sources in the public domain

	Pharmacokinetics data	Toxicity data	
		Dose and MTD	ADE and DLT
Goodman and Gilman’s *The Pharmacological Basis of Therapeutics* [14]			
Drug Combination Database (DCDB) [8]			
Certera Drug Interaction Database (DIDB^®^) [9]			
Transformer Database [12]			
UCSF-FDA TransPortal [11]			
PharmGKB^®^ [13]			
FDA Adverse Event Reporting System (FAERS) [6]			
DrugBank [10]			
ClinicalTrials.gov [1]			
NLM’s Daily Med [7] (Drug Labels)			
AACR/ASCO meeting abstracts [15, 16]			
PubMed			

Note: AACR, American Association of Cancer Research; ADE, adverse drug event; ASCO, American Society of Clinical Oncology; DLT, dose-limiting toxicity; FDA, United States Food and Drug Administration; MTD, maximum tolerable dose; PharmGKB**^®^**, Pharmacogenomics Knowledgebase; UCSF, University of California, San Francisco.

**Figure 1.**
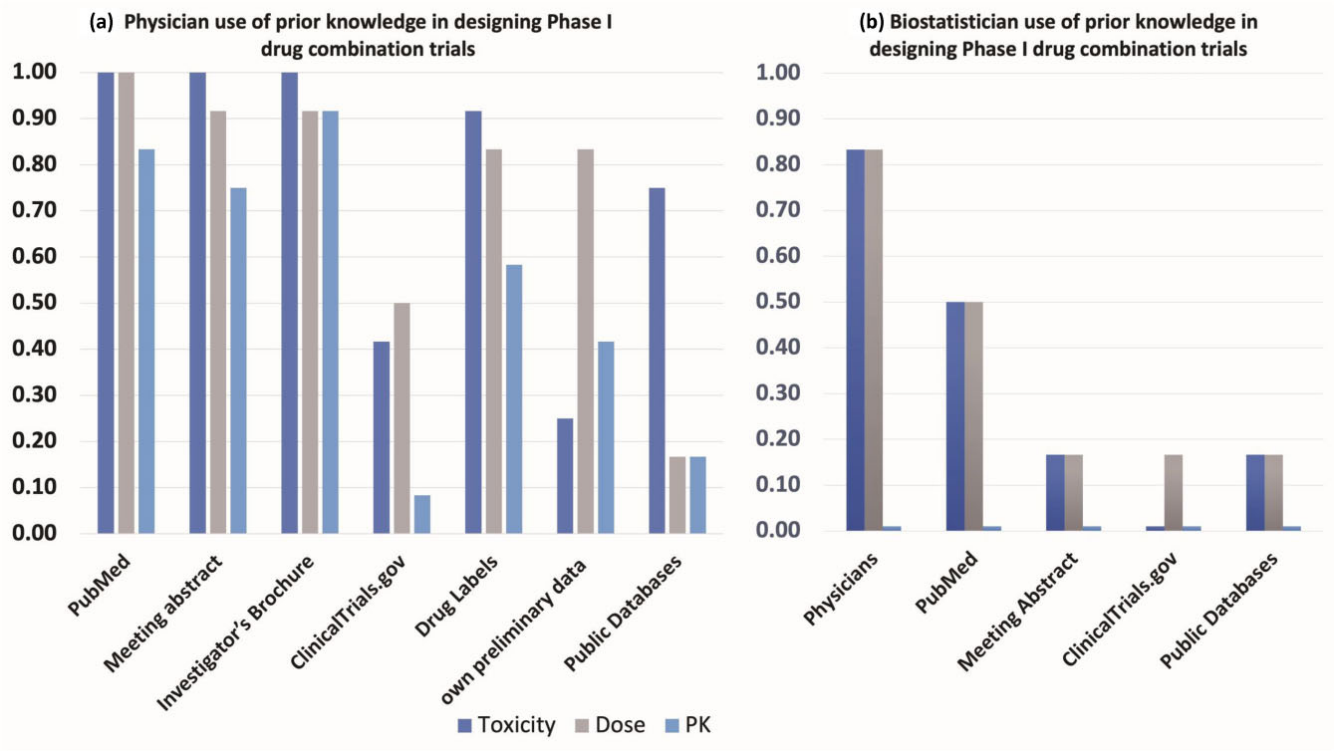
Results from an internal survey of *N* = 18 participants (12 physicians, 6 biostatisticians) from The Ohio State University Comprehensive Cancer Center (OSUCCC) clinical trial network and The Ohio State University Center for Biostatistics regarding use of prior knowledge in the design of Phase I trials of cancer drug combinations.

To gain preliminary insights and trends into resource utilization for designing Phase I drug combination trials within our local institutional context, we conducted a small internal survey at The Ohio State University Comprehensive Cancer Center and James Cancer Hospital (OSUCCC) and The Ohio State University Center for Biostatistics (Fig. 1). This survey involved 18 participants: 12 physicians actively involved in designing oncology clinical trials and 6 biostatisticians who collaborate on such trials.

We queried the physicians (Fig. 1a) about the specific resources they typically consult (including PubMed, meeting abstracts, investigator brochures, ClinicalTrials.gov, drug labels, internal preliminary data, and public domain databases) when gathering pharmacokinetics (PK), drug dose, and toxicity data for Phase I combination trial design. Among the 12 physicians surveyed, while most (>80%) reported using sources like PubMed, meeting abstracts, investigator brochures, and drug labels for dose and toxicity information, <20% reported searching public domain data sources specifically for PK data.

Separately, the six biostatisticians (Fig. 1b) were asked about the primary sources of their prior knowledge (PK, dose, toxicity) when contributing to these trial designs. The majority in this group (82%) indicated receiving dose and toxicity information primarily from physicians. Fewer reported obtaining information from PubMed (50%) or other resources like meeting abstracts, ClinicalTrials.gov, or public databases (18%). Notably, none of the biostatisticians surveyed explicitly mentioned PK data as information they typically received or considered from these sources.

To assist physicians and biostatisticians in designing anticancer combinational-drug Phase 1 trials, we developed DrugCombo, a comprehensive knowledge base that integrates PK, dosing, and toxicity data for both individual and combination drugs. DrugCombo provides up-to-date detailed information, including starting dose, dose range, and dose escalation intervals as well as other information, across various domains for the design of Phase 1 trials. Most importantly, DrugCombo is the exclusive repository of MTDs and DLTs from published Phase I clinical trial results.

## Materials and methods

### Manually curated maximum tolerant doses and dose-limiting toxicities from published Phase 1 clinical trial results

This section describes the manual curation process of MTDs and DLTs derived from published Phase 1 clinical trials. Data curation was guided by a tripartite knowledge model. Addtionally, the biocuration methodology included acquiring articles, providing curator instructions, conducting preliminary validation, and implementing a formalized procedure to ensure data integrity and consistency.

#### Clinical trial knowledge model

**Figure 2.**
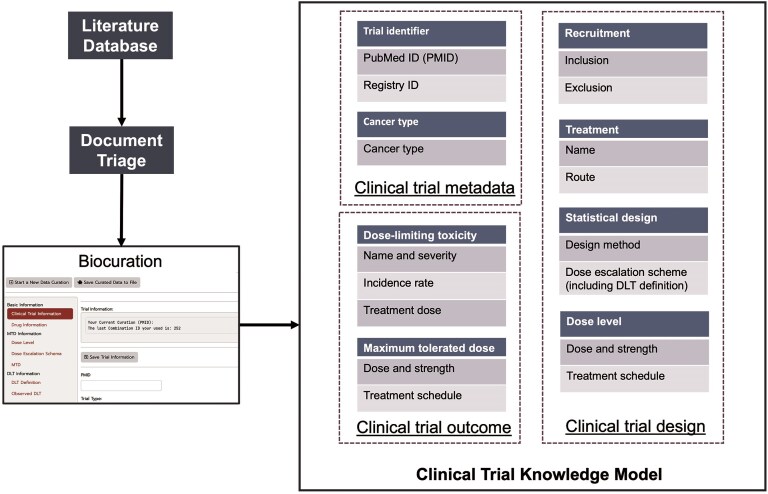
Procedure for the biocuration of data from published Phase I clinical trials.

We developed a three-component model of clinical trial knowledge (Fig. 2) for the curation of data from published Phase I clinical trials, which comprises clinical trial metadata, design data, and outcome data.

1. For the curated Phase I trials, clinical trial metadata define the trial and publication identifiers and specify the type and location of the cancer under investigation. Trial Identifiers represent the primary source of data and comprise two attributes, PubMed ID (PMID) and Registry ID, that narrow the data source. Because our current work focuses on the curation of publication data from PubMed, our primary key is the PMID. Registry identifiers are clinical trial registration IDs from several data sources, including cancer.gov, ClinicalTrials.gov, or the University Hospital Medical Information Network (UMIN) of Japan. The other data type, Cancer Type, indicates the specific cancer type/s under investigation in a clinical trial.
2. *Clinical trial design data* aim to capture the a priori information from the trial protocol. The Recruitment class defines the criteria for patient eligibility and exclusion, and curators collect age and gender information of the participants from the inclusion criteria of the trials. The Treatment class indicates information about cancer therapies, including small molecular drugs, therapeutic proteins, and radiotherapies, employed in clinical trials, and includes the drug name, formulation, and administration route. The Statistical Design class specifies the statistical methodology of the trial, such as the ‘3 + 3’ or Bayesian methods and dose escalation/de-escalation schemes. Moreover, the definitions of MTD and DLT are also curated. Dose is the other class independent of Treatment and includes the details of drug strength, doses, and treatment schedule for each evaluated dose in the clinical trial.
3. *Clinical trial outcome data* consist of MTD and DLT classes, which capture the trial’s empirical results, form the core of the entire clinical trial knowledge model and share similar attributes. The MTD class defines the maximum tolerated dose determined by the trial or dose recommended by the trial, and the DLT class characterizes the occurrence of DLT in the trial. For each DLT, attributes collected included the name of ADE, its severity grade, the associated treatment dose, and the observed frequency. Since DLTs are defined in trial protocols based on severity and presumed relationship to the study treatment, this curation captures the critical treatment-related toxicities identified in these Phase I studies.

#### Biocuration method

Figure 2 illustrates the overall biocuration workflow for data from published Phase I clinical trials. We retrieved candidate articles using a query set of keywords from PubMed via the E-Utilities tool of the NCBI [17]. Curators then examined the retrieved articles and classified them as relevant or irrelevant, and the set of anticancer drug Phase I trial-relevant articles was further curated in depth based on the data curation protocol. Finally, the curated knowledge was stored and organized per our clinical trial knowledge model. Five expert curators manually collected all data with machine assistance. To ensure data integrity, two curators with backgrounds in pharmacology (Dr Wang and Ms Zhang) initialized the data curation protocol development stage (Supplementary Materials S1) and conducted the first round of pilot curation testing, and three additional curators with strong statistical training (Drs Wei and Zhu and Ms Xia) then joined the data curation team after strict training. Dr Wei has extensive experience in Phase 1 trial design and was therefore given particular responsibility for judging and verifying any ambiguous trial design method.

*Document triage*: The first step of our biocuration pipeline was to triage publications and identify those relevant to Phase 1 clinical trials of anticancer drugs. We used the following PubMed query to retrieve potentially relevant articles: ((<anticancer drug name>) AND (‘MTD’[All Fields] OR ‘Phase 1’[All Fields] OR ‘RP2D’[All Fields])) AND (clinicaltrial[Filter]). From this query, we limited the article type to ‘Clinical Trial’ to obtain precise search results and then assigned the candidate articles to the five curators to review their relevance to Phase I clinical trials of anticancer drugs and removed those deemed irrelevant.
*Curator training and pilot testing*: To guarantee the consistency of curation standards among them, each biocurator was required to review the data curation protocol thoroughly and highlight any ambiguity and concerns for group discussion. A total of 10 papers were then distributed to curators for pilot testing.
  In the pilot testing stage, all the curators utilized the data curation tool to extract relevant knowledge from the test papers based on the data curation protocol, and the entire team validated the curated data, resolving any inconsistency through discussion in group meetings to achieve group consensus. The validation criteria were defined based on the Clinical Trial Knowledge Model (Table 2).
*Formal biocuration*: Relevant articles selected from the previous document triage step were sent to the entire biocuration team for data curation.

**Table 2.** Validation criteria in pilot testing

Data element	Criteria
Trial identifier	(a) The PMID should match the PubMed search record. (b) All the registry IDs in the paper should be extracted.
Cancer type	Cancer sites should be specified.
Eligibility criteria and exclusion criteria	(a) Eligibility criteria and exclusion criteria should be separated correctly. (b) Age and/or sex information should be curated from papers.
Treatment	(a) Drug names must be correct. Synonyms are allowed. (b) Therapy administration route and formulation should be curated.
Statistical design	(a) The statistical method should be selected correctly from the tool’s built-in list. (b) The dose escalation/de-escalation scheme should be specified and clear. (c) The definition of maximum tolerated dose (MTD) should be clear and correct. (d) The definition of dose-limiting toxicity (DLT) should contain adverse drug event (ADE) names, severity, and any necessary information.
Dose level	(a) The drug name should match with the Treatment class. (b) Dose and strength must be clear and correct. (c) The treatment schedule may contain daily treatment or scheduled treatment day in one cycle.
DLT	(a) DLT names and severity must be correct. (b) The dose at the time of the DLT should refer to the correct dose level or be specified. (c) The incidence rate should be computed based on the dose at the time of the DLT.
MTD	(a) The drug name should match with the Treatment class. (b) Dose and strength must be clear and correct. (c) The treatment schedule may contain daily treatment or treatment day in one cycle. (d) If the trial failed to determine an MTD, the reason should be annotated (e.g. MTD not reached). (e) If the trial finds the recommended dose for the Phase 2 trial (RP2D), the ‘RP2D’ term should be specified.

### Automatic extraction of adverse drug events from the structured product labels

The critical drug safety knowledge was available to the public in the form of structured product labels (SPLs), which contain ADE information in many sections, including Boxed Warnings, Warnings and Precautions, and Adverse Reactions. The Boxed Warnings section contained the severe drug-induced ADEs, whereas the Adverse Reactions section provided a landscape of commonly seen ADEs from the late-phase clinical trials. Such drug safety knowledge, however, remained in the free narrative text that was not computable and integrated with other knowledge.

Some studies have attempted to extract ADE knowledge from SPLs, but up-to-date information may be unavailable in data sources. For instance, the SIDER Side Effect Resource (SIDER) [18, 19], the largest repository of product labelling data, contains information on 1430 marketed medicines and associated ADEs from various public sources that include the FDA SPLs, but SIDER has not been updated since 2015 and does not therefore include the most recent drug safety knowledge regarding anticancer treatments.

In this study, we trained a pretrained large language model (LLM)—Microsoft Research (MSR) Bidirectional Encoder Representations from Transformers for Biomedical Text Mining (BiomedBERT; formerly PubMedBERT) [20]—to extract ADE information from SPLs automatically.

#### Training and evaluation dataset for LLM performance improvement

We used the SPL-ADR-200db dataset [10], created by Dr Dina Demner-Fushman of the Computational Health Research Branch of the National Library of Medicine (NLM), to train the LLM and evaluate model performance. The SPL-ADR-200db is a fine-grained manual annotation of the adverse reactions of SPLs by the collaborative effort of the NLM and the FDA. This dataset consists of 101 SPLs as the training set and 99 SPLs as the test set. In total, the entire dataset included 15 722 mentions, including 13 795 adverse reactions. For our ADE extraction task, we used only adverse reactions mentions to train the MSR BiomedBERT model to improve its capacity to recognize named entities.

#### Fine-tuning and evaluation of language model for ADE extraction

We fine-tuned the MSR BiomedBERT-large model [20] specifically for the ADE extraction task using the SPL-ADR-200db training dataset [21]. The model’s original tokenizer was used. Key hyperparameters for fine-tuning included a maximum sequence length set to 256 tokens, a batch size of 128, and a learning rate of 1e−5. The model was trained for 15 epochs using cross-entropy loss, which is appropriate for this token classification task. Training was conducted on a single server with an Intel^®^ Xeon^®^ CPU @ 2.00 GHz CPU, 32 GB RAM, and an NVIDIA^®^ V100 Tensor Core GPU.

To evaluate the performance of our fine-tuned model, we assessed its performance on the SPL-ADR-200db test set using the micro-averaged F1 score. Furthermore, we conducted a comparative analysis by fine-tuning and evaluating other widely used LLMs (including BERT [22], BioBERT [23], PharmBERT [24], and Clinical BERT [25]) under identical experimental conditions and using the same evaluation metric. To ensure robustness, the training and evaluation process for each model was replicated five times, and the average micro-averaged F1 scores, precision and recall were used for comparison.

The comparison of performance across all tested models are provided in the Supplementary Materials (Supplementary Fig. 1)

### Detection of significant ADE signals from the FAERS

We obtained postmarketing drug surveillance data from the FAERS database, which collected data for suspected ADEs for further analysis. In DrugCombo, we aimed to analyse the FAERS data and identify the associations between ADEs and individual drugs or two-drug combinations as another essential type of drug safety knowledge.

#### Processing FAERS data

We downloaded all the FAERS reports from 1 January 2004 through 30 September 2018. Because FAERS is a voluntary reporting system, its inclusion of nonstandardized drug names and duplicated cases significantly reduces the quality of its data. It is therefore necessary to address quality issues before analysis using FAERS data.

*Drug name normalization*: We first mapped all the drug names with RxNorm and then conducted additional exploratory analysis for the unmapped drug names. In this step, we removed uninformative parts from drug names, including drug form (e.g. tablet), strength (e.g. 10 mg) and pharmaceutical salt form (e.g. hydrochloride) and then utilized RxNorm again to capture drug brand names. We also used additional international brands from DrugBank. For the remaining unmapped drug names, we used the USAGI software tool [26] created by Observational Health Data Sciences and Informatics (OHDSI) for manual mapping.
*Case deduplication*: As recommended by the FDA, we performed a deduplication step to retain the most recent report for each case with the same case identifier. In our study, all the available cases were extracted based on the case ID, case initial/follow-up code (‘I’ or ‘F’), demographic information, prescribed drugs, and reported ADEs. If all these fields were the same, the most recent case version was kept as the unique case.
*ADE normalization*: FAERS utilizes the preferred terms (PT) of the Medical Dictionary for Regulatory Activities (MedDRA^®^) to describe all DDIs and ADEs. Lower-level terms (LLT) are also used in certain situations. We used the MedDRA^®^ PT for all the indication and ADE data fields, incorporating as well any LLTs previously mapped to PT terms. We then removed cases with matching terms for indications and adverse events, which might not represent a drug-induced ADE.

#### ADE signal detection

We utilized disproportionality analyses (DPAs) to detect statistically significant drug–ADE signals to provide physicians and biostatisticians with actionable knowledge for designing clinical trials. To ensure detection of all possible individual drug-induced ADEs in line with established practices for FAERS data, our statistical analysis was performed at the individual drug–ADE association level, without controlling for multiplicity across all drugs or all ADEs. Such DPAs compared the observed report frequency for a drug–ADE pair (observed frequency) to the baseline frequency under the assumption of no association (expected frequency) to quantify and prioritize the associations between drugs and ADEs. Two primary DPAs for individual drugs, the reporting odds ratio (ROR) [27] and proportional reporting ratio (PRR) [28], were applied in the current stage. Furthermore, DrugCombo reported the 95% confidence intervals (CI) of the ROR and PRR as the reference of knowledge strength.

Additionally, for the associations between drug–drug combinations and ADEs, we implemented a shrinkage observed-to-expected ratio statistical model to detect possible drug–drug–ADE signals [29]. Similarly, this method also investigated the associations by comparing the observed report frequency of a drug–drug–ADE combination to its baseline frequency, assuming no interactions between the two drugs. The shrinkage observed-to-expected ratio, denoted as omega (Ω), was calculated as the binary logarithm of the observed-to-expected ratio [29]. In our database, the threshold for Ω is set to 0, indicating the observed frequency of a specific ADE reported with the concomitant use of both drugs exceeds the frequency expected based on their independent reporting rates.

To focus the analysis on commonly reported events and ensure sufficient data frequency for potentially reliable pattern detection, the case reports were filtered based on the overall frequency of drugs and ADE terms within the dataset in our analysis. Specifically, only drugs appearing in at least 100 reports and ADE terms appearing in at least 1000 reports were included in the analyses.

### Curation of pharmacokinetics knowledge from literature and other sources

We derived the PK knowledge in the DrugCombo primarily from manual curation of published literature and external publicly available databases. In the current version, we focused on drug metabolism and elimination knowledge that could help researchers avoid potential DDIs.

The fraction of the dose excreted unchanged in urine (fe) was curated from the Biopharmaceutics Drug Disposition Classification System (BDDCS) [30]. The drug name, fe, and reference were curated as essential data elements. We only curated the quantitative parameters from the BDDCS for possible use in further model-based PK-DDI prediction. The clearance data, including drug name, clearance value, and clearance type (plasma, liver, or overall), were curated primarily from DrugBank.

The fractions of metabolism by cytochrome P450 (CYP450) enzymes (fm) were curated from published drug metabolism studies using the human liver microsome [31]. The fm was defined as the relative contributions of the CYP450 isozyme. Drug names, references, CYP450 enzymes and their corresponding inhibitors, study type (substrate depletion or metabolite formation), and fm value were essential to demonstrate the fractions of metabolism by CYP450.

### Data normalization and standardization

**Figure 3.**
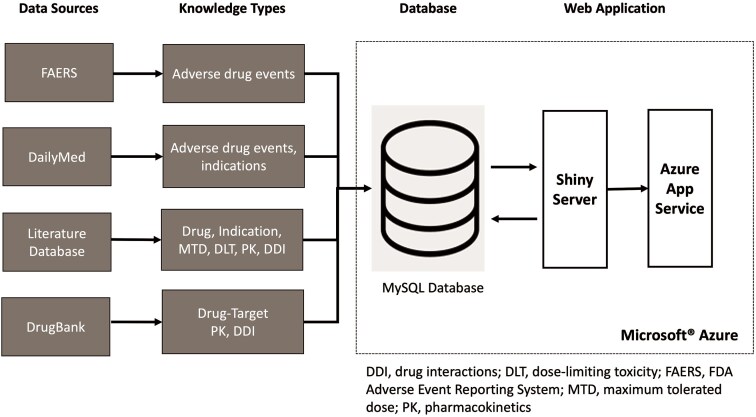
Integration of data from different sources into the DrugCombo knowledge base.

DrugCombo integrates knowledge from various resources utilizing different terminologies (Fig. 3). For example, DrugBank generally uses the DrugBank ID as the drug identifier to connect the generic name with other information, whereas drug names curated from publications may use generic names, brand names, and even some synonyms and abbreviations as identifiers. Thus, normalization and standardization of data must be carried out prior to its importation into the database to allow users to access all available information, creating a straightforward path to data discovery and analysis. In this study, we use the NLM’s Unified Medical Language System (UMLS) as the dictionary for data normalization and standardization.

The primary terminologies that should be normalized and standardized in the DrugCombo include drug names, disease names, including cancer types, and ADEs. We used a multiple-step process to standardize data from all sources. The following paragraphs outline the generic data-mapping process by illustrating the mapping of ADE terms because the mapping process varies for each data type, such as medications and disease, and is relatively similar from start to finish.

Step 1: *Exact mapping of terms by UMLS terminologies*: In DrugCombo, ADEs originate from multiple sources, both external resources (e.g. FAERS) and internal resources (e.g. biocuration from publications). External sources always utilize MedDRA^®^ terminology, enabling the direct mapping of these external data entities to the corresponding UMLS concept and controlled unclassified information (CUI) because UMLS has integrated MedDRA^®^ as one of the vocabularies. In handling the biocuration results, we first attempt an exact match with the strings in the UMLS dictionary.
Step 2: *MSR BiomedBERT-based ADE recognition and mapping*: Among those unmapped terms in Step 1, we used a fine-tuned LLM (MSR BiomedBERT) [20, 32] to recognize the ADEs. LLM fine-tuning was detailed in the section ‘Automatic extraction of adverse drug events from the structured product labels’. We then used MetaMap to map the recognized ADE terms to UMLS concept IDs.
Step 3: *Manual mapping*: The remaining unmatched data are fed into the manual matching/assignment step.

### DrugCombo knowledge base development

Figure 3 depicts the organization of DrugCombo with its fundamental database and web application. We utilized the Microsoft^®^ Azure Cloud infrastructure to host the database server (MySQL Flexible Server) and provide web service (Azure App Service). Our cloud-based architecture provided a scalable and reliable web application infrastructure; MySQL Community Server Version 8 served as the database engine, and the R Shiny framework and other necessary R packages were used to develop the entire web application.

#### Database design

We designed a robust drug-centric data model to store extracted knowledge for single- and combinational drugs in a structured format. This relational data model primarily integrated various data resources regarding clinical drug name/dose, toxicity name/frequency/severity, and patient population.

Table 3 summarizes the tables in the current database. The tables were categorized into two types—knowledge data and terminology. The knowledge data tables were named according to the types of knowledge stored in them and their source. For instance, a record in the table ‘db_ddi’ stored DDI knowledge from DrugBank.

**Table 3.** Tables in the current DrugCombo knowledge base

Table type	Category	Table name	Table content
Knowledge table	Drug–drug interaction	db_ddi	Evidence of drug–drug interaction from DrugBank
	Pharmacokinetics	fm	Fractions of metabolism by cytochrome P450 (CYP450) enzymes of individual drugs
		fe	Fraction of dose excreted unchanged in urine of individual drugs
		cl	Clearance of individual drugs
	Target	target	Targets of individual drugs
	Adverse events	fda_ade	Significant adverse drug event (ADE) signals detected from the Adverse Event Reporting System of the United States Food and Drug Administration (FAERS) data
		spl_ade	Severe and commonly seen ADEs extracted from drug labels
	Maximum tolerated dose (MTD) and drug-limiting toxicity (DLT)	trial_meta	Trial and publication identifiers, cancer group of the curated Phase I trials
		trial_design	Statistical design of the curated Phase I trials, including design methods, dose escalation scheme
		treatment	Detailed drug information, including name, dose, and administration route of the curated Phase I trials
		dose_level	Studied drug doses of the curated Phase I trials
		mtd	MTDs or recommended doses of the curated Phase I trials
		dlt	Observed DLTs from the curated Phase I trials
		dlt_definition	Detailed DLT definitions of the curated Phase I trials
Terminology table	-	umls	Standardized terms from the Unified Medical Language System (UMLS)

Generally, a unique table for each type of knowledge, including DDIs, drug safety, and PK, stored corresponding data. The drug concept ID, the critical standardized drug name terminology according to UMLS, served as the necessary foreign key to facilitate linking of records with other tables having the same drug. For example, the association of rows in the label_ade table and faers_ade table using the drug identifier could allow the assignment of all the ADEs to the queried drug. We also utilized multiple tables to manage the curated MTD and DLT knowledge because of the complexity of the knowledge model (described in the section ‘Manually curated maximum tolerant doses and dose-limiting toxicities from published Phase 1 clinical trial results’). For these tables, the PubMed ID was considered as a unique identifier to associate trials. The terminology tables are named according to the dictionary name, e.g. the ‘meddra’ table is used to normalize ADE terms by the MedDRA^®^ dictionary.

#### Development of the DrugCombo web application

The web application was implemented using the R Shiny framework; all visual orientation was optimized by the Bootstrap v5.0 framework on top of the Shiny framework, and fundamental and critical functionalities were supported by several additional R packages. For example, the Plotly and DT packages provided an interactive data visualization to end users.

## Results

### Database content and statistics

Fundamentally, DrugCombo is a drug-centric knowledge base intended for multiple purposes but strongly focused on aiding the design of Phase I clinical trials. It integrates various types of knowledge, including publicly available data-rich content, such as that in DrugBank, data mining results from FAERS and SPLs, and our own expert-curated knowledge (Fig. 3). We strive to close the existing knowledge gap in Phase I clinical trial design by consolidating various expertise into a cohesive and openly accessible informatics resource. This database can also support scientists of diverse backgrounds in identifying combinations of effective anticancer drugs.

DrugCombo currently contains 14 595 drug entries, 472 of which are anticancer drug entries, including 239 FDA-approved anticancer therapies. These drug entries include FDA-approved small-molecule drugs, FDA-approved biotech (protein/peptide) drugs, and experimental drugs. The knowledge in the current version of DrugCombo can be categorized into five classes: pharmacological knowledge, PK knowledge, adverse reaction knowledge, DDI knowledge, and clinical trial knowledge. Table 4 breaks down the statistics of knowledge in each class, including the associated numbers of drugs, drug pairs, and related publications.

**Table 4.** DrugCombo knowledge statistics

Category	Knowledge	Statistics
Target	Drug–target/gene interaction	8577 pairs
Drug–drug interaction (DDI)	DDI evidence from DrugBank	8797 pairs
Adverse drug event (ADE)	Severe ADE from drug labels	3995 pairs
	Common ADE from drug labels	95 535 pairs
	Significant ADE signals from the Adverse Event Reporting System of the United States Food and Drug Administration (FAERS)	1 816 030 pairs
MTD and DLT	Expert-curated Phase I trial data	2748 papers
Pharmacokinetics	Fraction of metabolized by cytochrome P (CYP) enzymes	42 drugs
	Hepatic clearance	195 drugs
	Fraction of dose excreted unchanged in urine	1319 drugs

#### Manually curated maximum tolerated dose and dose-limiting toxicity knowledge

The DrugCombo knowledge base includes 2748 published Phase I clinical trials of anticancer drugs that involved 472 anticancer agents and 52 cancer types. Of these curated trials, 13.2% (363) involved a single agent as treatment, and the remaining 86.8% (2385) involved multiple drugs. Our curated knowledge also demonstrates that most (>99%) of the Phase I clinical trials were designed using conventional ‘3 + 3’ methods.

#### Safety knowledge from SPLs

Supplementary Fig. S1 depicts results of the comparison of average test F1 scores among our fine-tuned MSR BiomedNLPBert model and other models. In the current experiment setting, the benchmark of best performance results was the score achieved by a long short-term model in the 2017 Text Analysis Conference (TAC 2017). Our fine-tuned MSR BiomedNLPBert model achieved an F1 score of 0.90 and recall of 0.91.

In the data extraction from SPLs, 50 421 human prescription labels are downloaded from DailyMed [7] in XML format. Currently, we do not include any over-the-counter products in the DrugCombo knowledge base. All these labels were parsed, and the Boxed Warnings sections were extracted using FDA Logical Observation Identifiers Names and Codes (LOINC) code 34066-1 and the Adverse Reactions sections using LOINC code 34084-4. Then, the fine-tuned MSR BiomedBERT model for ADE extraction (described in 2.4.1) was applied for prediction.

In total, we found 99 535 ADE terms in the Adverse Reactions sections and 3995 ADE terms in the Boxed Warnings sections over 1533 individual ingredients. We included only single-ingredient products in our knowledge base because of the clear relationship between ADE and the ingredient (drug).

#### Pharmacokinetics knowledge

DrugCombo contains the exclusive knowledge of contributions of 11 primary CYP enzymes involved in hepatic metabolism for 42 anticancer small-molecule drugs (Table 4). Other PK knowledge in the current version of DrugCombo includes the fraction of dose excreted unchanged in urine (fe) for 1319 drugs, including 230 FDA-approved anticancer drugs, and clearance data for 1638 drugs that were manually curated and verified from DrugBank. Such PK knowledge is highly valuable for clinical scientists to understand the absorption, distribution, metabolism, and excretion (ADME) properties of anticancer drugs and estimate dosage regimens when designing a drug combination trial.

#### Safety knowledge from FAERS

After drug-name mapping and removal of duplicate reports, a total of 8 888 579 case reports from FAERS were imported into our database for further analysis. We filtered the case reports based on the frequency of individual drug and ADE terms (frequency threshold for single drugs, 99, and for ADR terms, 999). After preprocessing, a total of 5787 unique drugs, associated with 19 919 unique MedDRA^®^ terms and yielding 5 150 178 drug–ADE pairs, were identified.

After the FAERS database-wide screening for individual drugs and two-drug combinations, we applied the following criteria to determine the evidence for significant ADE signals. The lower bound of the 95% CI of the PRR or ROR should meet the threshold of two for individual drugs, and for two-drug combinations, combination–ADE associations should meet the threshold disproportional reporting rate of Ω > 0.

In the DrugCombo knowledge base, 1948 307 (37.82%) significant drug–ADE pairs were identified according to PRR, and 1 947 661 (37.81%) significant drug–ADE pairs were identified according to ROR. Furthermore, database-wide screening using the omega shrinkage method found 343 950 drug–drug–ADR triplets with Ω > 0.

### Data access and web application

DrugCombo can be accessed using a web browser at https://drugcombo.info. Because the primary goal of DrugCombo is to provide an informatics tool for efficient design of Phase 1 clinical trials, the current web application is designed to display preferentially knowledge that relates to clinical trial design, such as MTD, DLT, and safety profiles.

#### Search function of DrugCombo web application

**Figure 4.**
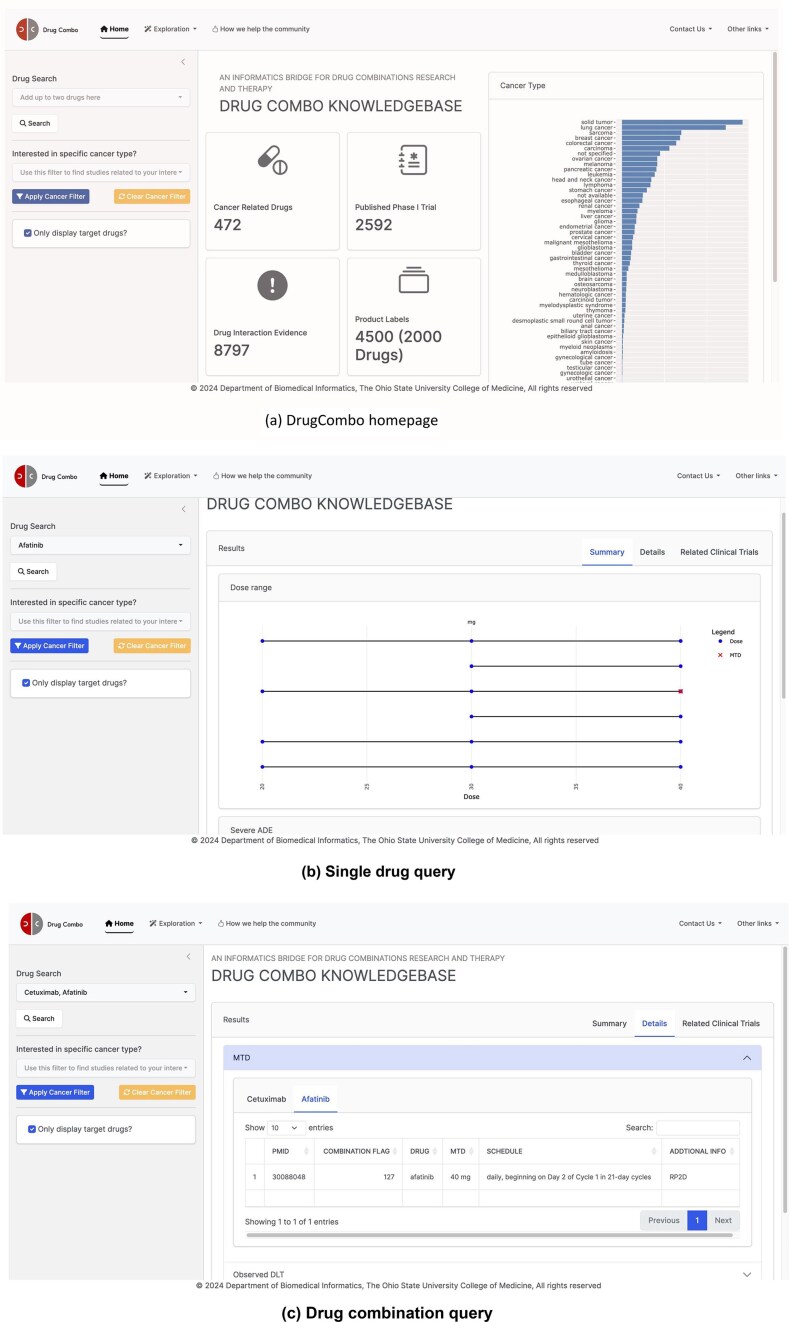
DrugCombo data portal: (a) DrugCombo homepage, (b)Single drug query result page, and (c) Drug combination query result page.

As a drug-centric knowledge base, DrugCombo supports standard text queries (through the text search box located on the home page, Fig. 4). Users can use one drug entry to obtain the knowledge for an individual drug or two drugs for individual and combinational knowledge. The search box supports partial matching, allowing users to type parts of the generic name, brand name, or synonyms and select the drug from a drop-down list. To customize the results, two types of filters are provided. Users can refine the cancer type and display only information regarding the search drug(s).

For knowledge that is not directly related to clinical trial design, DrugCombo offers an information-browsing function using the ‘Exploration’ buttons located at the top of each page (Fig. 4a). For specific browsing, summary tables provide drug targets, anatomical therapeutic chemical classification (ATC) codes, and mechanism-of-action (MoA) information, and all the summary tables can be rapidly searched and sorted.

#### Knowledge representation in DrugCombo

We provide search results using a summary page and a details page. The summary page presents crucial individual and combinational data objectively. A dumbbell plot illustrates the evaluated doses and MTD in each trial, with curated values grouped by reported units. This type of plot clearly illustrates the drug-dose knowledge and can assist researchers in choosing the starting dose for a Phase I clinical trial. The summary page also provides essential drug safety knowledge. For individual drugs, DrugCombo provides a summary table that lists all the severe ADEs mentioned in the Boxed Warning section of SPLs so that clinical scientists and biostatisticians can promptly ascertain the serious risks. For the drug-combination query, we utilized a table to summarize the overlapping ADEs between the two drugs, information we believe is critical when designing a combinational trial. In addition, for combination search, DDI evidence can also be provided on the summary page.

The detail page is designed to present more detailed knowledge, which is organized into summary tables. For example, users can explore the detailed extracted ADEs from SPLs for each component of their queried combination. The detail page also lists detailed DLTs and corresponding MTDs, including treatment schedules of curated Phase 1 trial results. Moreover, we provide a separate page (called ‘Clinical Trial information’) so that users can obtain the detailed trial design and other metadata of their interest. Users can click the PubMed ID column in MTD or DLT Tables to access the detailed design information.

### Demonstrating the utility of DrugCombo knowledge for Phase I trial design of anticancer combinational drugs: nivolumab and axitinib in patients with advanced renal cell carcinoma

In this case study, we utilized a completed Phase I/II trial from ClinicalTrials.gov (NCT03172754) to demonstrate how the knowledge in the DrugCombo can assist in the design of Phase I trials of anticancer combinational drugs.

#### Original trial design

Axitinib, a small molecule and tyrosine kinase inhibitor, is used to treat advanced renal cell carcinoma, whereas nivolumab, a biologic antibody and PD-1 inhibitor, was approved in 2014 for the treatment of various types of cancer. In the Phase I part of its original study design, which utilized a standard ‘3 + 3’ design to evaluate three doses of axitinib along with a standard fixed dose of nivolumab, 6–12 patients with advanced renal cell carcinoma were assessed to determine the recommended Phase II dose of anxitinib. The starting 3.0-mg dose of axitinib could be escalated to 5.0 mg or de-escalated to 2.0 mg with the 480.0-mg dose of nivolumab based on dose-limiting toxicities. DLTs were defined as severe haematologic ADEs, including neutropenia, febrile neutropenia, neutropenic infection, and thrombocytopenia with or without bleeding, and severe nonhaematologic ADEs, including nausea, vomiting, diarrhoea, iritis, episcleritis, eye pain, blurred vision, and hypertension. All the DLTs occurred within the 28-day assessment window.

#### Existing drug safety profiles from DrugCombo

**Figure 5.**
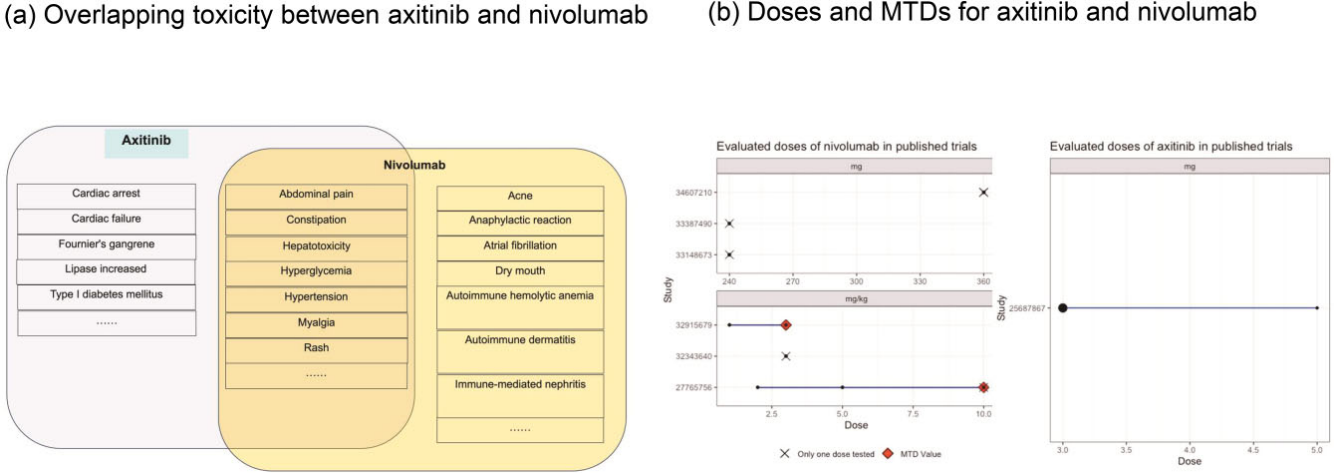
Example of Phase I trial design using DrugCombo: (a) Overlapping toxicity between axitinib and nivolumab and (b) Doses and maximum tolerated doses (MTDs) for axitinib and nivolumab.

Using axitinib and nivolumab as a combination input in DrugCombo, the Summary tab indicates there are no severe overlapping ADEs between the two drugs (Fig. 5a). However, there are some common overlapping ADEs, including hypertension, anaemia, and hyperglycaemia. The detailed ADE knowledge from drug labels indicated that the toxicity profile of nivolumab (Fig. 5a) attributed most nivolumab-induced ADEs to immune-related ADEs, whereas axitinib could probably induce cardiovascular and haemal events.

#### Potential pharmacokinetic DDI between two drugs

No PK DDI evidence was provided in DrugCombo. As a small molecular drug, axitinib is metabolized primarily by CYP3A4/5 and, to a lesser extent, by CYP1A2, CYP2C19, and UGT1A1, while the therapeutic protein nivolumab is mostly degraded in targeted tissues other than the liver [33].

#### Maximum tolerated doses for individual drugs

In total, DrugCombo provides curated results of six Phase 1 trials of nivolumab and one trial of axitinib. The curated trials evaluated nivolumab at a fixed dose [34–36] (240 and 360 mg) or in a range from 1.2 to 10.0 mg/kg [37, 38] and investigated axitinib in a range of 3.0 to 5.0 mg. Nivolumab was found to have an MTD at 3.0 and 10.0 mg/kg in two trials, which is 210.0 mg and 700.0 mg for an average person. Moreover, axitinib is tolerated at the maximum dose of 5.0 mg (Fig. 5b).

#### Potential trial design of combined axitinib and nivolumab using existing knowledge

Knowledge provided by DrugCombo demonstrates minimal interaction and no severe drug safety issue between axitinib and nivolumab. Thus, a dose of 5.0 mg for axitinib and 3.0 mg/kg for nivolumab could potentially achieve an optimum combination dose with therapeutic effects while ensuring patient safety.

This Phase I study could be designed as a Phase Ib study to start at a 5.0-mg maximum dose of axitinib with dose reduction to 3.0 and 2.0 mg if needed and maintenance of a 3.0-mg/kg dose of nivolumab based on guidelines. The period for evaluating DLT remained the same for the first cycle. The definition of DLT could include any Grade 3 or higher nonhaematologic toxicity, any Grade 4 haematologic toxicity that does not resolve within 48 hours, any Grade 2 diarrhoea or colitis that does not resolve within 48 hours with supportive care, any Grade 3 colitis or diarrhoea, or any Grade 3 hypertension. This would allow for an expected DLT rate of 25% at the MTD, based on the high rate of hypertension with axitinib, which would not be expected to be exacerbated by nivolumab.

## Discussion and conclusion

Despite increasing numbers of ongoing clinical trials of anticancer drug combinations that highlight the promising trend towards multiagent cancer therapies, many Phase I clinical trials of combinational drugs fail. Incorporating prior knowledge regarding the properties of both single drugs and their combinations into the design of Phase I trials may facilitate the selection of appropriate doses and schedules to avoid severe or life-threatening ADEs, improve patient safety, and lead to successful trial outcomes.

DrugCombo is intended to provide a high-quality, integrated, and comprehensive knowledge source for individual drugs and drug combinations to assist clinical oncologists and biostatisticians investigating multiagent cancer therapies. We provide a user-friendly tool for investigators to relate evidence from different aspects of anticancer drugs. The current version of DrugCombo provides 8577 drug–target interaction and drug mechanisms of action, a fraction of metabolized-by-CYP enzymes for 42 drugs, 8797 pieces of DDI evidence, and >90 000 drug–ADE pairs. In particular, 2592 published Phase I trial results have been curated for MTD and DLT knowledge.

DrugCombo is unique among publicly available drug databases that can be used to facilitate the design of Phase I trials of combinational drugs, offering a uniform platform that normalizes and integrates knowledge from such external specialized databases as the UCSF-FDA Transportal and SIDER. Furthermore, we also note that some essential knowledge for Phase I clinical trial design, including MTD and DLT, is missed in the current comprehensive databases, DrugBank and DrugCentral, for example. So, as one approach to rectify this deficit, we curated 2592 published Phase 1 trial results from the literature in PubMed and established a well-designed protocol to curate data based on a comprehensive knowledge model to capture the most complete knowledge possible and thereby assure data quality and reproducibility. Our annotators have strong pharmacological and statistical backgrounds and undergo training and pilot testing prior to data curation.

Our study has several limitations. Although the current DrugCombo knowledge base includes drug safety profiles that reflect both clinical trial experience and postmarketing surveillance, there is no standardized and quantitative tool for evaluating the toxicity of a potential combination. More specifically, incorporating the quantitative severity and prevalence of ADEs can provide more stratified and detailed information to help define accurate dose-limiting toxicities. As well, the inclusion of data regarding ADE severity and prevalence in specific subpopulations, such as elderly, maternal, and paediatric populations, may enhance the drug safety profile and facilitate the design of Phase 1 trials in particular populations. Moreover, current drug safety knowledge in DrugCombo primarily represents only drugs approved by regulation. The knowledge base cannot help in predicting the safety of drugs still in the preclinical phase. Considering 89.2% of the ongoing trials of drug combinations in ClinicalTrials.gov contain new drugs, a feasible approach to expand DrugCombo coverage is to include preclinical study evidence for drug toxicity.

Additionally, the current DrugCombo focuses primarily on clinical knowledge, integrating very limited knowledge of target pathways and functional annotations that enable investigations of target upstream/downstream effects. In the future, we will expand DrugCombo with molecular knowledge to provide new insights into drug combinations at the molecular level.

In summary, the DrugCombo knowledge base is a comprehensive public repository of anticancer drug data that provides high-quality, manually curated, and unique MTD and DLT knowledge, integrating valuable knowledge from external publicly available sources to assist researchers in designing efficacious Phase 1 trials. We expect this knowledge base to become a hub for the investigation of multiagent anticancer therapies, a valuable resource for drug discovery and development.

## Supplementary Material

baaf043_Supplemental_Files

## Data Availability

DrugCombo is freely available online at https://drugcombo.info. The dataset repository can be accessed at https://github.com/langli-lab/drugcombo-data

## References

[1] National Library of Medicine . ClinicalTrials.gov. Date accessed 25 April 2024.

[2] Wong CH, Siah KW, Lo AW. Estimation of clinical trial success rates and related parameters. Biostatistics. 2019;20:273–86. 10.1093/biostatistics/kxx069 29394327 PMC6409418

[3] van Brummelen EM, Huitema AD, van Werkhoven E, et al. The performance of model-based versus rule-based phase I clinical trials in oncology: a quantitative comparison of the performance of model-based versus rule-based phase I trials with molecularly targeted anticancer drugs over the last 2 years. J Pharmacokinet Pharmacodyn. 2016;43:235–42. 10.1007/s10928-016-9466-0 26960536

[4] Chow SC . Adaptive clinical trial design. Annu Rev Med. 2014;65:405–15. 10.1146/annurev-med-092012-112310 24422576

[5] Lin R, Yin G. Bayesian optimal interval design for dose finding in drug-combination trials. Stat Methods Med Res. 2017;26:2155–67. 10.1177/0962280215594494 26178591

[6] US Food and Drug Administration . openFDA. https://open.fda.gov. Date accessed 01 November 2023.

[7] National Library of Medicine . DailyMed. https://www.dailymed.nlm.nih.gov/dailymed/index.cfm. Date accessed 24 April 2024. 10.1080/15360280801989377 28792816

[8] Liu Y, Wei Q, Yu G, et al. DCDB 2.0: a major update of the drug combination database. Database. 2014;2014:bau124. 10.1093/database/bau124 25539768 PMC4275564

[9] Certara . Drug Interaction Database (DIDB^®^). https://www.druginteractionsolutions.org/solutions/drug-interaction-database/. Date accessed 25 April 2024.

[10] Wishart DS, Feunang YD, Guo AC, et al. DrugBank 5.0: a major update to the DrugBank database for 2018. Nucleic Acids Res. 2018;46:D1074–82. 10.1093/nar/gkx1037 29126136 PMC5753335

[11] Morrissey KM, Wen CC, Johns SJ, et al. The UCSF-FDA TransPortal: a public drug transporter database. Clin Pharmacol Ther. 2012;92:545–46. 10.1038/clpt.2012.44 23085876 PMC3974775

[12] Hoffmann MF, Preissner SC, Nickel J, et al. The transformer database: biotransformation of xenobiotics. Nucleic Acids Res. 2014;42:D1113–17. 10.1093/nar/gkt1246 24334957 PMC3965107

[13] Whirl-Carrillo M, Huddart R, Gong L, et al. An evidence-based framework for evaluating pharmacogenomics knowledge for personalized medicine. Clin Pharmacol Ther. 2021;110:563–72. 10.1002/cpt.2350 34216021 PMC8457105

[14] Brunton LL, Knollmann BC. Goodman & Gilman’s the Pharmacological Basis of Therapeutics. New York, NY: McGraw Hill Medical, 2022, p. 1.

[15] American Society of Clinical Oncology . Abstracts for the 2023 annual meeting. https://meetings.asco.org/abstracts-presentations/. Date accessed 25 April 2024.

[16] American Association for Cancer Research .Abstract Presentations for AACR annual meeting 2023 .https://www.aacr.org/meeting/aacr-annual-meeting-2023/abstracts/. Date accessed 25 April 2024.

[17] National Center for Biotechnology Information (US) . Entrez Programming Utilities Help. 2010. https://www.ncbi.nlm.nih.gov/books/NBK25501 10.3163/1536-5050.98.2.012 PMC2859267 20428285

[18] Kuhn M, Campillos M, Letunic I, et al. A side effect resource to capture phenotypic effects of drugs. Mol Syst Biol. 2010;6:343. 10.1038/msb.2009.98 20087340 PMC2824526

[19] Kuhn M, Letunic I, Jensen LJ, et al. The SIDER database of drugs and side effects. Nucleic Acids Res. 2016;44:D1075–79. 10.1093/nar/gkv1075 26481350 PMC4702794

[20] Tinn R, Cheng H, Gu Y, et al. Fine-tuning large neural language models for biomedical natural language processing. Patterns. 2023;4:100729. 10.1016/j.patter.2023.100729 37123444 PMC10140607

[21] Demner-Fushman D, Shooshan SE, Rodriguez L, et al. A dataset of 200 structured product labels annotated for adverse drug reactions. Sci Data. 2018;5:180001. 10.1038/sdata.2018.1 29381145 PMC5789866

[22] Devlin J, Chang M-W, Lee K, et al. Bert: pre-training of deep bidirectional transformers for language understanding. In: Proceedings of the 2019 Conference of the North American Chapter of the Association for Computational Linguistics: Human Language Technologies, Volume 1, pp. 4171–86. Minneapolis, MN: Association for Computational Linguistics, 2019.

[23] Lee J, Yoon W, Kim S, et al. BioBERT: a pre-trained biomedical language representation model for biomedical text mining. Bioinformatics. 2020;36:1234–40. 10.1093/bioinformatics/btz682 31501885 PMC7703786

[24] ValizadehAslani T, Shi Y, Ren P, et al. PharmBERT: a domain-specific BERT model for drug labels. Briefings Bioinf. 2023;24:bbad226. 10.1093/bib/bbad226 37317617

[25] Liu X, Liu H, Yang G, et al. A generalist medical language model for disease diagnosis assistance. Nat Med. 2025;31:932–42. 10.1038/s41591-024-03416-6 39779927

[26] Observational Health Data Sciences and Informatics (OHDSI) team . USAGI. https://github.com/OHDSI/Usagi. Date accessed 01 December 2022.

[27] Evans SJ, Waller PC, Davis S. Use of proportional reporting ratios (PRRs) for signal generation from spontaneous adverse drug reaction reports. Pharmacoepidemiol Drug Saf. 2001;10:483–86. 10.1002/pds.677 11828828

[28] van Puijenbroek EP, Bate A, Leufkens HG, et al. A comparison of measures of disproportionality for signal detection in spontaneous reporting systems for adverse drug reactions. Pharmacoepidemiol Drug Saf. 2002;11:3–10. 10.1002/pds.668 11998548

[29] Noren GN, Sundberg R, Bate A, et al. A statistical methodology for drug–drug interaction surveillance. Stat Med. 2008;27:3057–70. 10.1002/sim.3247 18344185

[30] Bocci G, Oprea TI, Benet LZ. State of the art and uses for the Biopharmaceutics Drug Disposition Classification System (BDDCS): new additions, revisions, and citation references. AAPS J. 2022;24:37. 10.1208/s12248-022-00687-0 35199251 PMC8865883

[31] Hua L, Chiang CW, Cong W, et al. The cancer drug fraction of metabolism database. CPT: Pharmacomet Syst Pharmacol. 2019;8:511–19. 10.1002/psp4.12417 PMC6656935 31206254

[32] Gu Y, Tinn R, Cheng H, et al. Domain-specific language model pretraining for biomedical natural language processing. ACM Trans Comput Healthcare. 2021;3:1–23.

[33] Zientek MA, Goosen TC, Tseng E, et al. In vitro kinetic characterization of axitinib metabolism. Drug Metab Dispos. 2016;44:102–14. 10.1124/dmd.115.065615 26512042

[34] Gutierrez M, Moreno V, Heinhuis KM, et al. OX40 agonist BMS-986178 alone or in combination with nivolumab and/or ipilimumab in patients with advanced solid tumors. Clin Cancer Res. 2021;27:460–72. 10.1158/1078-0432.CCR-20-1830 33148673

[35] O’Hara MH, O’Reilly EM, Varadhachary G, et al. CD40 agonistic monoclonal antibody APX005M (sotigalimab) and chemotherapy, with or without nivolumab, for the treatment of metastatic pancreatic adenocarcinoma: an open-label, multicentre, phase 1b study. Lancet Oncol. 2021;22:118–31. 10.1016/S1470-2045(20)30532-5 33387490

[36] Clarke JM, Patel JD, Robert F, et al. Veliparib and nivolumab in combination with platinum doublet chemotherapy in patients with metastatic or advanced non-small cell lung cancer: a phase 1 dose escalation study. Lung Cancer. 2021;161:180–88. 10.1016/j.lungcan.2021.09.004 34607210

[37] Fukuoka S, Hara H, Takahashi N, et al. Regorafenib plus nivolumab in patients with advanced gastric or colorectal cancer: an open-label, dose-escalation, and dose-expansion phase Ib trial (REGONIVO, EPOC1603). J Clin Oncol. 2020;38:2053–61. 10.1200/JCO.19.03296 32343640

[38] Apolo AB, Nadal R, Girardi DM, et al. Phase I study of cabozantinib and nivolumab alone or with ipilimumab for advanced or metastatic urothelial carcinoma and other genitourinary tumors. J Clin Oncol. 2020;38:3672–84. 10.1200/JCO.20.01652 32915679 PMC7605393

