# Supplementary material for: DrugCombo: an informatics bridge for anticancer drug combination Phase I trial design
Source: Database (Oxford). 2025 Sep 24;2025:baaf043. doi: 10.1093/database/baaf043 (PMC12462634; doi:10.1093/database/baaf043)
Supplement: baaf043_Supplemental_Files [file baaf043_supplemental_files.zip › S1.pdf]

MTD/DLT data curation protocol

Forked from NCBI data curation protocol

Max Wong

M Max Wong

Protocol Info: Max Wong . MTD/DLT data curation protocol. **protocols.io** **https://protocols.io/view/mtd-dlt-data-curation-protocol-b3ieqkbe**

Created: January 05, 2022

Last Modified: April 11, 2022

Protocol Integer ID: 56614

Keywords: curation, Drug combo, MTD, DLT, clinical trial

Disclaimer

Please note that this protocol is public domain, which supersedes the CC-BY license default used by protocols.io.

Clinical Trial Information Curation

1 **The Clinical Trial Information Curation protocol details how to collect clinical trial information from the published Phase 1 trial results.**

1.1 **PMID : Pubmed ID (PMID)** is used as a primary key to identify a specific clinical trial . There are two ways to retrieve a PMID:
(a) Search with title in the PubMed
(b) Look for Zotero: In ***Extra*** of **Info** section

Info | Notes | Tags | Attachments | Related

[illegible]	[illegible]
Author	Ruppert, Amy S.
Author	Byrd, John C.
Author	Culler, Kristy
Author	Wilkins, Diedre
Author	Wright, John J.
Author	Grever, Michael R.
Author	Shapiro, Charles L.
Publication	Cancer Chemotherapy and Pharmacology
Volume	66
Issue	1
Pages	151-158
Date	2010-05
Series	
Series Title	
Series Text	
Journal Abbr	Cancer Chemother Pharmacol
Language	eng
DOI	10/fq387v
ISSN	1432-0843
Short Title	
URL	
Accessed	
Archive	

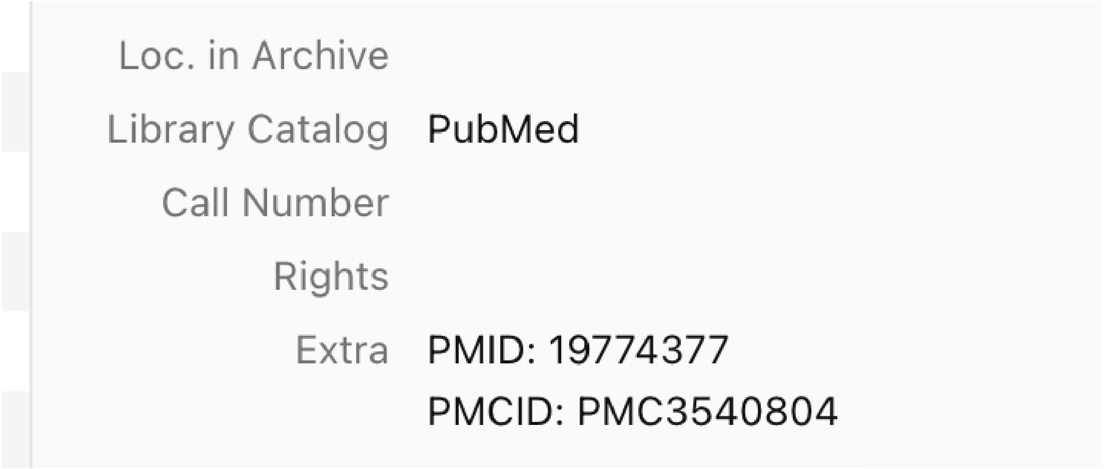

1.2 **Registered ID:** Many clinical trials were registered in a database (e.g., clinicaltrials.gov). The most common Registered ID is NCT ID. Search in the full text and the abstract using **free text** that starts with **nct.**
Otherwise, a Registered ID could be found at the end of the **Introduction** section or the footnote on the first page.

Invest New Drugs (2015) 33:1187–1196
DOI 10.1007/s10637-015-0278-7

PHASE I STUDIES

A first-in-human phase I dose-esc
and pharmacodynamic evaluation
a glycogen synthase kinase 3 inhib
in combination with pemetrexed a

Jhanelle E. Gray[1] · Jeffrey R. Infante[2] · Les H. Brail[3,6] · G
Jennifer F. Cooksey[1] · Suzanne F. Jones[2] · Daphne L. Farri
Kimberley A. Jackson[3] · Kay H. Chow[3] · Maciej J. Zamek-
Howard A. Burris III[2]

Received: 1 May 2015 / Accepted: 11 August 2015 / Published online: 25 S
© Springer Science+Business Media New York 2015

Summary *Purpose* LY2090314 (LY) is a glycogen synthase kinase 3 inhibitor with preclinical efficacy in xenograft models when combined with platinum regimens. A first-in-human phase 1 dose-escalation study evaluated the combination of LY with pemetrexed/carboplatin. *Patients and Methods* Forty-one patients with advanced solid tumors received single-dose LY monotherapy lead-in and 37 patients received LY (10–120 mg) plus pemetrexed/carboplatin (500 mg/m^2 and 5–6 AUC, respectively) across 8 dose levels every 21 days. Primary objective was maximum tolerated dose (MTD) determination; secondary endpoints included safety, antitumor activity, pharmacokinetics, and

Presented at the American Society of Clinical Oncology Annual Meeting; Chicago, IL; June 3–7, 2011. J Clin Oncol 29 (suppl): abstract 3030.

Registered under ClinicalTrials.gov (http://clinicaltrials.gov) identifier: NCT01287520.

✉ Howard A. Burris, III
howard.burris@scresearch.net

[1] H. Lee Moffitt Cancer Center and Research Institute, Tampa, FL, USA

[2] Sarah Cannon Research Institute and Tennessee Oncology, 250 25th

1.3 **Disease population:** The following information may be captured from the paper:

(a) cancer type*
(b) age
(c) gender
(d) race
(e) socioeconomic status
(f) comorbidities
(g) disease severity

Cancer type is the required information while others are optional. You could find cancer types in the title.

Note: For those general cancer types (e.g., solid tumors), the specific cancer types recruited in the trial can be found in the **patients**, **study design**.

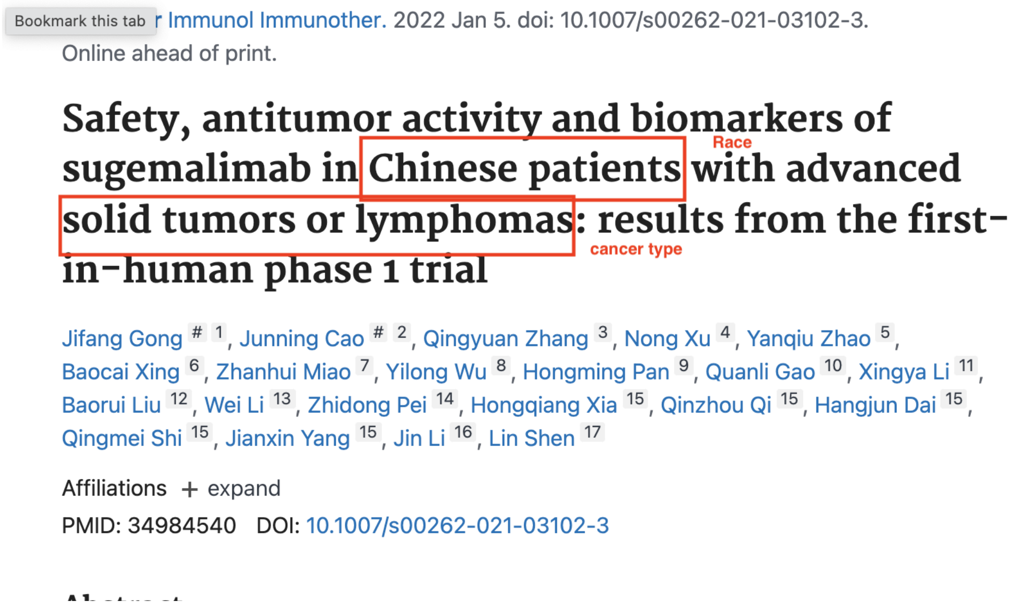
Bookmark this tab r Immunol Immunother. 2022 Jan 5. doi: 10.1007/s00262-021-03102-3.
Online ahead of print.

Safety, antitumor activity and biomarkers of sugemalimab in Chinese patients with advanced solid tumors or lymphomas: results from the first-in-human phase 1 trial

Jifang Gong [#, 1], Junning Cao [#, 2], Qingyuan Zhang [3], Nong Xu [4], Yanqiu Zhao [5], Baocai Xing [6], Zhanhui Miao [7], Yilong Wu [8], Hongming Pan [9], Quanli Gao [10], Xingya Li [11], Baorui Liu [12], Wei Li [13], Zhidong Pei [14], Hongqiang Xia [15], Qinzhou Qi [15], Hangjun Dai [15], Qingmei Shi [15], Jianxin Yang [15], Jin Li [16], Lin Shen [17]

Affiliations + expand

PMID: 34984540 DOI: 10.1007/s00262-021-03102-3

1.4 Inclusion and Exclusion: This information can be found in the Patients subsection. Some papers also provide full definitions in the supporting information.

Patients

Patients were recruited from 2 study centers in phase 1a and 15 study centers in phase 1b in China. Eligible patients were 18–75 years old; had histologically or cytologically confirmed unresectable, locally advanced or metastatic solid tumors or lymphomas with at least one measurable/evaluable lesion according to Response Evaluation Criteria in Solid Tumors version 1.1 (RECIST v1.1) (solid tumors) or Lugano Classification 2014 (lymphomas); progressed since previous standard anti-cancer therapy; and had an Eastern Cooperative Oncology Group performance status (ECOG PS) of 0–1. Key exclusion criteria included known primary central nervous system (CNS) tumors; prior malignancy other than those specified in phase 1b within the past 5 years; and major cardiovascular diseases. Full inclusion and exclusion criteria are listed in Supplementary materials. All patients provided written informed consent; study procedures were approved by an independent ethics committee at each study center.

1.5 **DLT Evaluation Period:** A time slot where an ADE was observed can be considered as a DLT. DLT Evaluation Period can be found by the end of the **Patients** subsection or in the **study design (e.g., the first cycle)**.

Drug information curation

2 The following drug information should be curated from the paper:
(a) Drug name
(b) Administration Route
(c) Dose Form
(d) **Drug Combination Components:** Each drug combination should be assigned with a combination ID. Sometimes there could be multiple drug combinations studied in a single trial. The curator should read carefully to identify the combinations. The annotation tool could assign an ID to the first combination automatically. Data curators could assign IDs to the following combinations they encounter manually.

2.1 **Drug Names**: The following lists some common types of drugs in the literature:

- Generic name: e.g., Gemcitabine
- Brand name: e.g., Gemzar (brand name of gemcitabine)
- Other synonym: e.g., Gemcitabin (Germain name of gemcitabine); 2',2'-Difluorodeoxycytidine (chemical name of gemcitabine)
- Experimental name: e.g., BNT162b2 (BNT-Pfizer covid vaccine). Some papers published early phase 1 trial results. Drugs in such papers were in the format of internal names (letter plus numbers)
- Abbreviated name: e.g., PTX (an abbreviated name for Pentoxifylline). A data curator may find abbreviations for cancer drug combinations e.g., AC refers to the combination of Doxorubicin Hydrochloride and Cyclophosphamide.

- Radiotherapy: radiotherapy should be captured as a drug name.

Safety information

Some Abbreviations for cancer drug combinations:

- ABVD: **Doxorubicin Hydrochloride**(A); **Bleomycin(B); Vinblastine Sulfate**(V); **Dacarbazine** (D)
- AC: **Doxorubicin Hydrochloride**(A); **Cyclophosphamide**
- ADE: **Cytarabine**(A); **Daunorubicin Hydrochloride**(D); **Etoposide Phosphate**(E)
- BEACOPP: **Bleomycin**; **Etoposide Phosphate**; **Doxorubicin Hydrochloride**; **Cyclophosphamide**; **Vincristine Sulfate**(O); **Procarbazine Hydrochloride**; **Prednisone**
- BEP: **Bleomycin**; **Etoposide Phosphate**; **Cisplatin**(P)
- CAF: **Cyclophosphamide**; **Doxorubicin Hydrochloride**(A); **Fluorouracil**
- CAPOX: **Capecitabine**(CAP); **Oxaliplatin**(OX)
- CEM: **Carboplatin**; **Etoposide Phosphate**; **Melphalan Hydrochloride**
- CHOP: **Cyclophosphamide**; **Doxorubicin Hydrochloride**(H); **Vincristine Sulfate**(O); **Prednisone**
- CMF: **Cyclophosphamide**; **Methotrexate**; **Fluorouracil**
- COPDAC: **Cyclophosphamide**; **Vincristine Sulfate**(O); **Prednisone**; **Dacarbazine**(DAC)
- COPP: **Cyclophosphamide**; **Vincristine Sulfate**(O); **Procarbazine Hydrochloride**; **Prednisone**
- CVP: **Cyclophosphamide**; **Vincristine Sulfate**; **Prednisone**
- EPOCH: **Etoposide Phosphate**; **Prednisone**; **Vincristine Sulfate** (O); **Cyclophosphamide**; **Doxorubicin Hydrochloride**(H)
- FEC: **Fluorouracil**; **Epirubicin Hydrochloride**; **Cyclophosphamide**
- FOLFIRI: **Leucovorin Calcium** (FOL); **Fluorouracil**; **Irinotecan Hydrochloride**(IRI)

2.2 **Dose form and Administration route**: These pieces of information could be primarily found in the **Study design** section. It could sometimes be found in the **Introduction** section.

- The authors may use an adverb to specify the administration route, e.g., orally. The data curator should read the related sections carefully.
- Sometimes, distinct dose forms of the same drug were evaluated in the trial. Thus, the data curator should differentiate the combinations with the corresponding dose forms.
- Differentiate the combinations and clarify the corresponding dose forms in the comments cell.

Dose Escalation Schema curation

3 Dose Escalation Schema information involves the following:

- **Design Type:** The most commonly used method for a Phase 1 trial is a **3+3 dose** escalation design. We also provide other options in the tool. The data curator should specify that the trial is escalation or de-escalation.
- **Dose escalation schema:** This info depicts how the trial steps into the next dose level or how the investigator determines the MTD (see Example below).

Example:

3.1 Design type information could be captured in the **Study Design** or **Statistical analysis** section.

This open label, multicenter, phase Ia/Ib study of DCDS0780A was designed to evaluate the safety, tolerability, pharmacokinetics, and recommended phase II dose (RP2D) according to a 3+3 dose escalation scheme. The phase Ia portion evaluated DCDS0780A monotherapy in

Safety information

If the data curator can not find study design method, just consult it with Dr. Lai Wei.

3.2 An example of dose escalation schema:

peared as a promising candidate to be used in combination with bevacizumab. This latter intravenous agent

the combination in patients with advanced renal cell carcinoma or with other advanced tumors. MTD was

Négrier *et al. BMC Cancer* (2017) 17:547 Page 3 of 10

defined as the highest dose level (DL) at which less than two of nine patients experienced a dose-limiting toxicity (DLT) during the first 8 weeks. Secondary ob- [illegible]

Treatment and dose escalation plan

Patients received oral pazopanib (Votrient®) (Novartis, Rueil-Malmaison, France) once daily at a dose of 400, [illegible]

Figures can be used to describe schema as well. The data curator could summarized the related information from the figure and input into the tool.

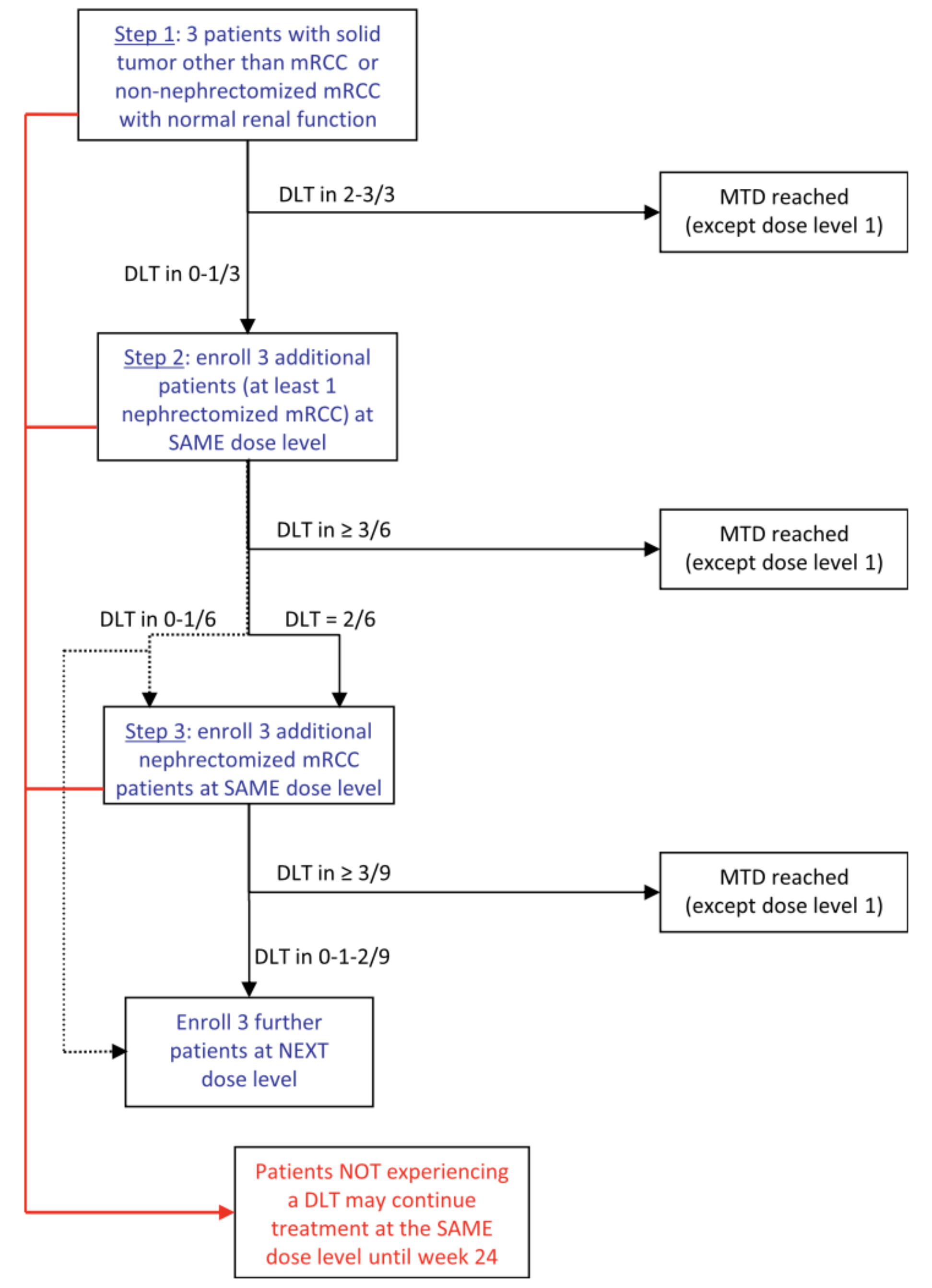

Fig. 1 Study schedule (3 + 3 + 3 escalation steps)

Dose levels curation

4 **Dose levels are critical in trial design. They may exist in:**
(a) a table
(b) a figure
(c) a paragraph of narrative

Typically, the data curator can find such information in the Study Design section.

Pleasem note: The starting dose, the dose chosen to treat the first cohort of patients in a phase I trial, typically has the dose level 0 or 1.

4.1 **Dose levels information in the table (example a)**

Table 1 Dose escalation of lenalidomide in combination with bevacizumab (28-day cycle)

Dose level	Lenalidomide PO (mg) daily × 21 days	Bevacizumab IV (mg/kg) every 2 weeks	Enrollment (N)	Completed cycle 1 (N)	DLT (N)
1	10	5	4	3	0
2	10	7.5	3	3	0
3	20	7.5	3	2	0
4[a]	20	10	21	19	0
Total			31	27	0

PO orally, *DLT* dose-limiting toxicity

[a] Including expansion phase

The first three columns in the table demonstrate the dose levels in this trial. For the drug combination, you can specify "fixed dose" in the Comment column if the drug was not escalated. (See 4.3, example c)

4.2 1. **Dose levels information in the figure (example b):**

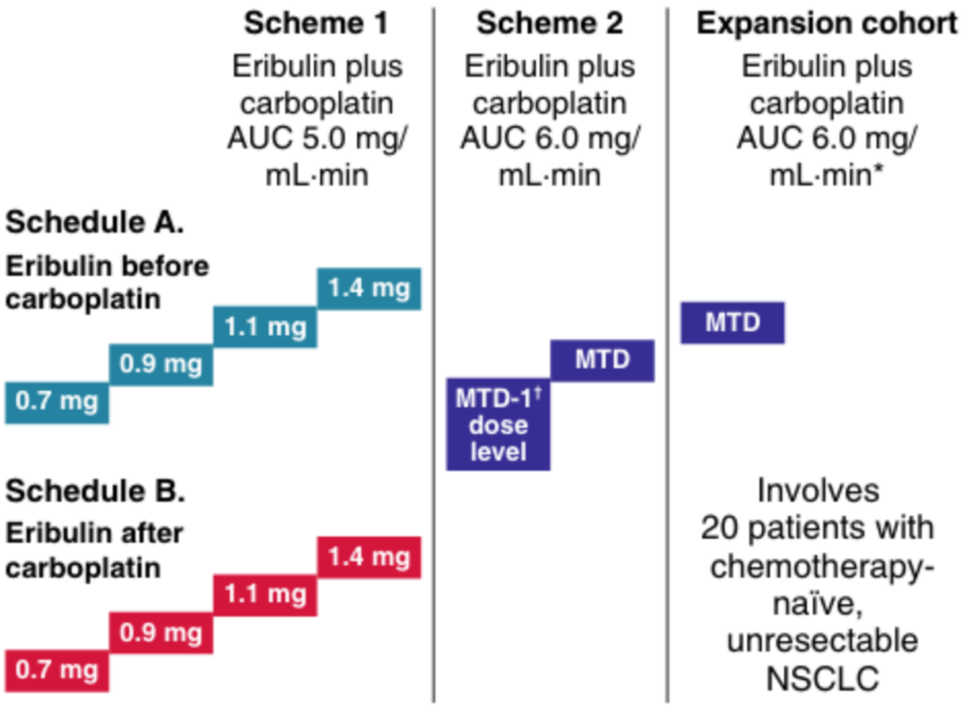

*If carboplatin AUC 6 is not tolerated, then an additional 20 patients will be enrolled at the MTD obtained with carboplatin AUC 5 plus eribulin.

†MTD-1 = 1 level below MTD.

3 Patients are enrolled into each dose level. If none of the 3 patients experience dose-limiting toxicities, 3 patients will be treated at the next dose level. However, if a DLT occurs in 1 of those 3 patients, 3 additional patients (total of 6) will be treated at that dose level.

Safety information

Sequence info could be captured and filled in the Comments column. (See example b)

4.3 **Dose levels information in the narrative (example c)**

(Detroit, Mich). Patients received oral buparlisib once daily in a continuous schedule of 28-day cycles. Bevacizumab was administered intravenously every 2 weeks. Buparlisib was administered at escalating doses (60 mg, 80 mg, and 100 mg once daily). Bevacizumab was administered at a fixed dose of 10 mg/kg. Once the MTD was [illegible]

A	B

5 **The authors typically use some sentences to define DLTs in the Study Design, Study assessments, or Statistical analysis section as well as supplementary material. DLT definitions may appear in the Statistical Considerations and Toxicity Evaluation Section as well.**

[illegible]

Adverse events (AEs) were graded according to the National Cancer Institute Common Terminology Criteria for Adverse Events, version 3.0. DLTs were measured in cycle 1 and were defined as nonhematologic or hematologic. Nonhematologic DLT included grade ≥ 3 toxicity (excluding untreated nausea, vomiting, tumor flare, or tumor lyses controllable by aggressive palliative therapy), which resolved to grade 2 or lower toxicity within 14 days. Hematologic DLTs included grade 4 throm[illegible]grade 3 thrombocytopenia with clinically significant bleeding, grade 4 neutropenia not reversible to grade 3 or better in ≤ 5 days without growth factor support, febrile neutropenia, or neutropenia associated with bacteremia or sepsis. Anemia or lymphopenia of any grade was not considered a DLT.

[illegible]

Safety information

Note: There may exist some exceptions for DLT definitions (e.g., Anemia or lymphopenia of any grade was not considered a DLT). Please curate and fill in the forms.

6 The data curator would find MTD information in the

(a) Abstract

(b) Results section

(c) Conclusion / Discussion

When MTD is not reached, established, or determined, the data curator should clarify it in the comments column.

If RP2D is reported instead of MTD, fill in the RP2D and label it in the comments column.

Example:

eribulin 1.4 mg/m^2 on Schedule B. No patient receiving this dose in Schedule A experienced a DLT (Table 2). Thus, we defined the MTD as 1.4 mg/m^2, and the optimum sequence of administration as eribulin followed by carboplatin (Schedule A). This dose and schedule were therefore used for the subsequent dose-escalation and expansion cohorts.

In the carboplatin AUC 6 cohort, DLTs occurred in 1 of 6 patients (febrile neutropenia) at the dose level of 1.1 mg/m^2, and in 2 of 3 patients (febrile neutropenia, neutropenia) at 1.4 mg/m^2 (Table 2). Thus, the MTD of eribulin in combination with carboplatin AUC 6 was 1.1 mg/m^2. This dosing regimen was subsequently used for the NSCLC-expansion cohort.

Safety information

As seen in the above example, please capture the critical additional info as well: e.g., Schedule, The dose for expansion cohort, etc.

7 Observed DLT:

Observed DLTs could be found in the Toxicity subsection of Results. They may exist in

(a) table

(b) figure

(c) narratives

Safety information

1. Please distinct AEs and DLTs.

DLTs are specific and pre-defined AEs in the study.

2. Please capture the dose level where DLTs occurred. (see 7.1)

7.1 Example: Observed DLT Table

Table 2 DLTs during cycle 1 in patients treated with eribulin + carboplatin AUC 5 or AUC 6

DLT	Eribulin dose and schedule							
	0.7 mg/m^2		0.9 mg/m^2		1.1 mg/m^2		1.4 mg/m^2	
	A	B	A	B	A	B	A	B
Carboplatin AUC 5, dose-escalation phase	**(*n* = 6)**	**(*n* = 6)**	**(*n* = 6)**	**(*n* = 8)**	**(*n* = 6)**	**(*n* = 3)**	**(*n* = 3)**	**(*n* = 5)**
Patients with DLT	1	1	1	1	1	0	0	1
Grade 4 febrile neutropenia					1			
Grade 4 neutropenia		1[a]						
Grade 4 thrombocytopenia	1							
Grade 3 leukopenia		1[a]						
Grade 3 arthralgia			1[a]					
Grade 3 neuralgia			1[a]					
Grade 3 hyperglycemia				1				
Grade 3 diarrhea								1
Carboplatin AUC 6, dose-escalation phase					**(*n* = 6)**		**(*n* = 3)**	
Patients with DLT					1		2	
Grade 4 febrile neutropenia					1			
Grade 3 febrile neutropenia							1	
Grade 4 neutropenia							1	
Carboplatin AUC 6, NSCLC cohort					**(*n* = 12)**			
Patients with DLT					4			
Febrile neutropenia, grade 3–4					4[b]			
Thrombocytopenia, grade 3–4					3[c]			
Grade 4 leukopenia					1			
Grade 3 gastroenteritis					1			

Schedule A, eribulin then carboplatin; Schedule B, carboplatin then eribulin

AUC area under the curve, *AUC 5* carboplatin AUC 5.0 mg/mL·min, *AUC 6* carboplatin AUC 6.0 mg/mL·min, *DLT* dose-limiting toxicity, *NSCLC* non-small cell lung cancer

[a] 1 Patient experienced 2 DLTs

[b] Febrile neutropenia was grade 4 (*n* = 2) or grade 3 (*n* = 2)

[c] Thrombocytopenia was grade 4 (*n* = 2) or grade 3 (*n* = 1)

8
