# Supplementary figures and images for: DrugCombo: an informatics bridge for anticancer drug combination Phase I trial design
Source: Database (Oxford). 2025 Sep 24;2025:baaf043. doi: 10.1093/database/baaf043 (PMC12462634; doi:10.1093/database/baaf043)

## Slide 1
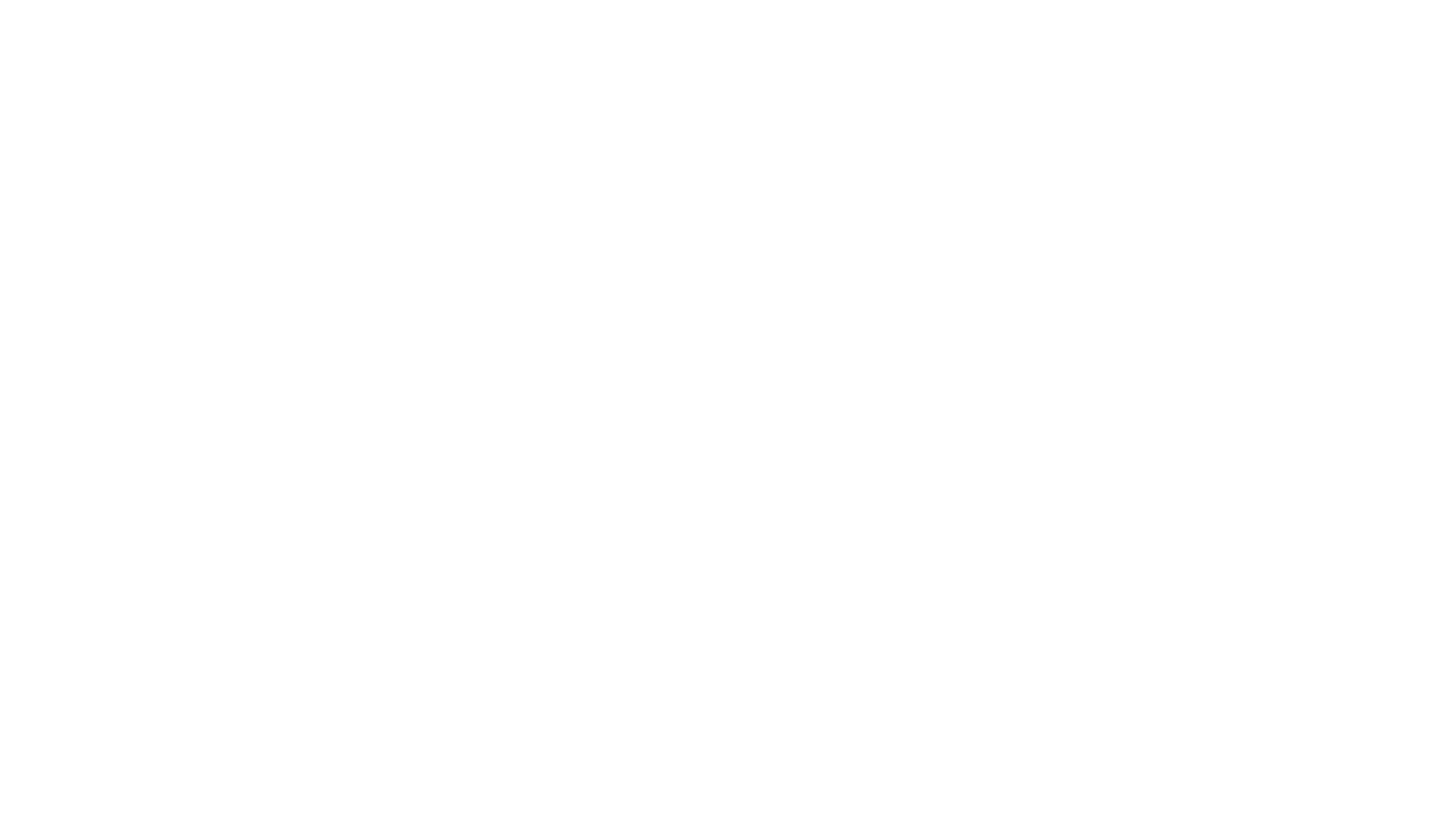

Supplement: baaf043_Supplemental_Files [file baaf043_supplemental_files.zip › FigureS1.pptx]
